# Supplementary material for: All-at-once RNA folding with 3D motif prediction framed by evolutionary information
Source: Nat Methods. 2025 Oct 3;22(10):2094–106. doi: 10.1038/s41592-025-02833-w (PMC12510878; doi:10.1038/s41592-025-02833-w)
Supplement: Supplementary file 3 — Source code to run CaCoFold-R3D. Data and results. [file 41592_2025_2833_MOESM3_ESM.gz › supplemental_material/results/CaCoFold-R3D/CaCoFold-R3D_Rfam/RF02046_Sphinx_1.R2R.sto.pdf]

RF02046_Sphinx_1

5′-R C G ● G R U G ● C A U U Y G G U G A C U A U C C A G ● U G A G U A C A A C C C C A A G G U G C A C G G G C C C U A ● G A C C C C G C U C G C U U C U A C G G Y A A A G ● Y ● R G C R G R Y ● ● C G Y A
